# Cost-Effectiveness of Salt Substitution and Antihypertensive Drug Treatment in Chinese Prehypertensive Adults

**DOI:** 10.1161/HYPERTENSIONAHA.124.23412

**Published:** 2024-10-28

**Authors:** Zhijia Sun, Haijun Zhang, Yinqi Ding, Canqing Yu, Dianjianyi Sun, Yuanjie Pang, Pei Pei, Ling Yang, Yiping Chen, Huaidong Du, Weijie Hu, Daniel Avery, Junshi Chen, Zhengming Chen, Liming Li, Jun Lv

**Affiliations:** Department of Epidemiology and Biostatistics (Z.S., Y.D., C.Y., D.S., Y.P., L.L., J.L.), School of Public Health, Peking University, Beijing, China.; Department of Health Policy and Management (H.Z.), School of Public Health, Peking University, Beijing, China.; Department of International Health, Bloomberg School of Public Health, Johns Hopkins University, Baltimore, MD (H.Z.).; Peking University Center for Public Health and Epidemic Preparedness and Response, Beijing, China (C.Y., D.S., Y.P., P.P., L.L., J.L.).; Key Laboratory of Epidemiology of Major Diseases (Peking University), Ministry of Education, Beijing, China (C.Y., D.S., L.L., J.L.).; Clinical Trial Service Unit and Epidemiological Studies Unit, Nuffield Department of Population Health, University of Oxford, United Kingdom (L.Y., Y.C., H.D., D.A., Z.C.).; Maiji Center for Disease Control and Prevention, Gansu, China (W.H.).; China National Center for Food Safety Risk Assessment, Beijing, China (J.C.).; State Key Laboratory of Vascular Homeostasis and Remodeling, Peking University, Beijing, China (J.L.).

**Keywords:** antihypertensive agents, Chinese, cost-effectiveness analysis, prehypertension, salt

## Abstract

**BACKGROUND:**

Recent guidelines recommend antihypertensive drug treatment for prehypertensive individuals with blood pressure between 130/80 and 139/89 mm Hg. This study evaluates the cost-effectiveness of 3 interventions in Chinese prehypertensive adults: salt substitution, antihypertensive drug treatment, and their combination.

**METHODS:**

We developed a Markov cohort model to estimate cardiovascular disease (CVD) events, costs, and quality-adjusted life years (QALYs) over a lifetime. Data from the China Kadoorie Biobank informed the simulation. Costs and utilities were drawn from published sources. We evaluated the cost-effectiveness of salt substitution alone, antihypertensive drug treatment alone, and a combination of the 2, focusing on the overall prehypertensive population, those at high CVD risk, and different starting ages (40, 50, 60, and 70 years). Incremental cost-effectiveness ratios (ICERs) were calculated per QALY gained.

**RESULTS:**

Salt substitution at age 40 years is the only cost-effective strategy for prehypertensive individuals, with an ICER of $6413.62/QALY. For those at high CVD risk, the combination intervention starting at age 40 years is most cost-effective, with an ICER of $2913.30/QALY. Interventions initiated at younger ages yielded greater CVD reductions and lower ICERs. For example, a combined intervention at age 40 years reduces CVD events by 5.3% with an ICER of $2913.30/QALY, compared with 4.9% and $32 635.33/QALY at age 70 years. These results were consistent across sensitivity analyses.

**CONCLUSIONS:**

In China, replacing usual salt with a salt substitute is more cost-effective than treating prehypertensive individuals over the age of 40 years with antihypertensive drugs. Furthermore, starting intervention at a younger age in prehypertensive adults can result in even greater cost savings.

**NOVELTY AND RELEVANCE**

**What Is New?**

- We evaluate the cost-effectiveness of 3 intervention strategies in Chinese prehypertensive adults: salt substitution alone, antihypertensive drug treatment alone, and a combination of the 2.

**What Is Relevant?**

- Salt substitution is a cost-effective strategy for the prehypertensive population over the age of 40 years. The most cost-effective strategy for prehypertensive adults at high risk of cardiovascular diseases is salt substitution intervention plus antihypertensive treatment. Interventions are more cost-effective when starting at an earlier age.

**Clinical/Pathophysiological Implications?**

- The findings indicate the benefit of salt substitution in prehypertensive adults and provide evidence for choosing the appropriate primary intervention strategy in prehypertensive adults in China.

High systolic blood pressure (SBP) is the leading risk factor for global attributable deaths, particularly cardiovascular deaths.^1^ Effective primary prevention and control measures could significantly reduce the global disease burden. It was estimated that about 19% of global deaths were attributed to high SBP (≥110–115 mm Hg) in 2019.^2^ In 2017, the American College of Cardiology/American Heart Association updated hypertension guidelines, lowering the threshold for hypertension from 140/90 to 130/80 mm Hg.^3^ Despite this revision, the threshold of 140/90 mm Hg is still widely used in major hypertension guidelines worldwide.^4–6^ However, some guidelines recommend a medication treatment for individuals with a blood pressure of 130–139/80–89 mm Hg, which is defined as prehypertension.^2,6,7^

Observational studies have demonstrated that prehypertension increases the risk of death and cardiovascular disease (CVD) events.^8,9^ A few randomized controlled trials (RCTs) have shown that early intervention in prehypertensive individuals reduced the risk of hypertension and CVD events effectively, including salt substitution intervention and antihypertensive drug treatment.^10–12^ Few studies have investigated the cost-effectiveness of these interventions in the prehypertensive population. Given the high prevalence of prehypertension in China,^13^ it is important to evaluate what measures should be taken for prehypertensive individuals from a health-economic perspective. To date, 3 studies have evaluated the cost-effectiveness of antihypertensive drugs in prehypertensive adults in China,^14–16^ but there is a notable gap in economic evaluations of salt substitution in this population. Furthermore, no study has thoroughly compared these 2 interventions or their combined approach.

This study aimed to evaluate the costs, effectiveness, and incremental cost-effectiveness ratio (ICER) of various intervention measures on Chinese prehypertensive adults, including antihypertensive drug treatment, salt substitution, and a combination of the 2. We seek to offer valuable insights and guidance for future policies addressing the management of prehypertension and health insurance in China.

## Methods

### Data Availability

The authors declare that most supporting data are available in Table 1. The China Kadoorie Biobank (CKB) data that support the findings of this study are available from the corresponding author upon reasonable request.

**Table 1.**
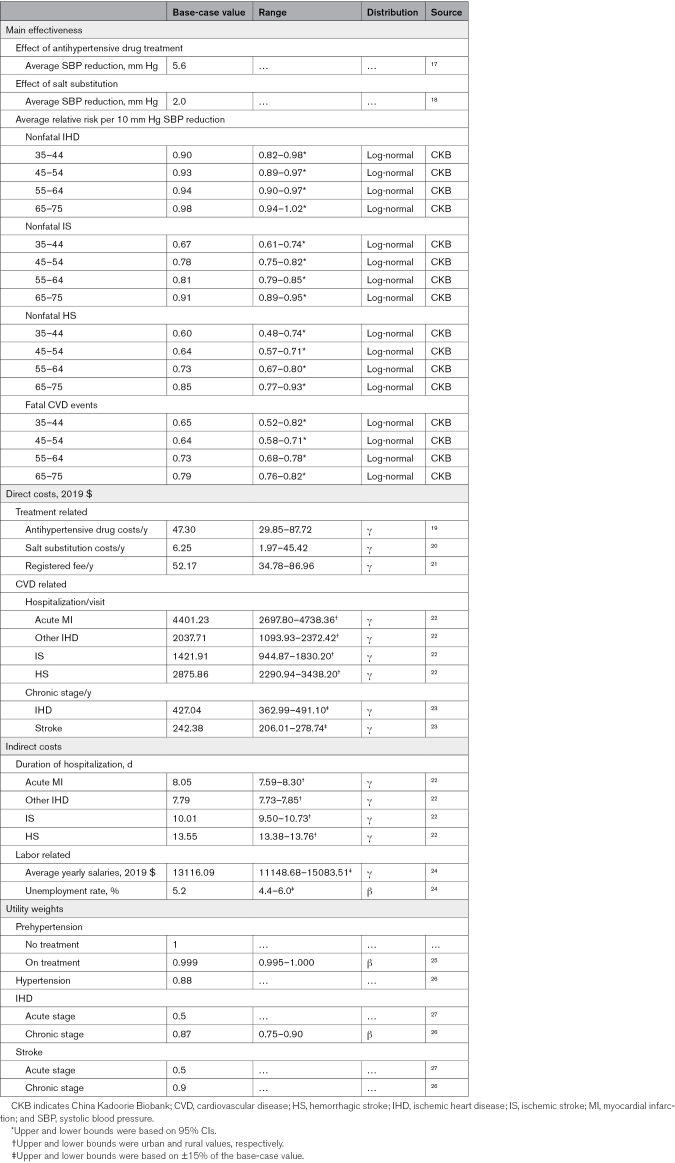
Parameters Used in the Cost-Effectiveness Model

### Model Overview

We developed a decision-analytic Markov cohort model to assess the cost-effectiveness of interventions for prehypertensive individuals from a societal perspective. Using a lifetime horizon, the model forecasted the incidence of CVD events, associated costs, and quality-adjusted life years (QALYs). This model was constructed on the foundation of the natural progression of CVD events, drawing upon previously established Markov models.^25,28^ The health states in the Markov model included disease-free state, nonfatal ischemic heart disease (IHD), nonfatal ischemic stroke, nonfatal hemorrhagic stroke, fatal CVD, and fatal non-CVD outcomes. Given the substantial cost differences in hospitalization between acute myocardial infarction and other IHD events, we separated these 2 nonfatal IHD events into 2 distinct states. The possible transitions between these states are depicted in Figure S1. Individuals without CVD at baseline will enter the model and experience the first nonfatal CVD event or fatal event. Nonfatal CVD events were divided into the acute stage (during hospitalization) and the chronic stage (posthospitalization). Recognizing the possibility of CVD comorbidity, individuals who undergo an initial nonfatal CVD event may experience recurrent CVD events. The shifts between nonfatal CVD events are reciprocal. Those who have nonfatal CVD events may advance to fatal CVD or non-CVD death, which are terminal states.

Each health state in this model received an annual cost and utility valuation. All costs were discounted to 2019 US dollars (US$1=6.9 RMB) and adjusted for inflation where necessary.^29^ The cohort simulation used data from the CKB study, with additional model parameters sourced from published literature (Table 1). Both costs and effectiveness were discounted at a rate of 5%, with the incremental cost per QALY gained serving as the primary outcome measure.^30^ In the absence of a specific cost-effectiveness threshold policy from the Chinese government, the threshold for evaluating the cost-effectiveness of interventions was set to the national gross domestic product (GDP) per capita for 2019 (US$ 10264.8), following the current Chinese guideline.^24,30^ The model was constructed using TreeAge Pro 2022 software (TreeAge Software, Williamstown, MA).

### Overview of the CKB Study

Data from the CKB study were used to simulate the Markov cohort model. The CKB is a large-scale prospective cohort study that enrolled 512 723 participants aged 30 to 79 years from 10 different regions of China, including 5 urban and 5 rural areas, between 2004 and 2008. The study areas were selected to ensure diverse risk factors and disease patterns, as well as high-quality death and disease registries, population stability, and local commitment. Details on the study design, procedures, and participant sociodemographics have been extensively reported elsewhere.^31^ In brief, each participant completed a detailed questionnaire via laptop, administered by an interviewer, while also undertaking physical measurements and providing blood samples at the baseline. This was conducted by professionally trained health workers after obtaining written informed consent from all participants.

Participants in the CKB underwent follow-up procedures that integrated both passive and active strategies, including linkage with death and disease registries as well as health insurance databases. All cases were meticulously coded by staff who were blinded to the baseline data, using the *International Classification of Diseases, 10th Revision* coding system. For the purposes of this study, CVD outcomes were specifically identified as myocardial infarction (I21–I23), IHD (I20–I25), hemorrhagic stroke (I61), and ischemic stroke (I63), with IHD events further categorized into acute myocardial infarction (I21) and other IHD events (I20, I22–I25). The rate of loss to follow-up remained <1% till December 31, 2018.

### Intervention Strategies

Three intervention strategies were investigated in this study for prehypertensive individuals: salt substitution alone, antihypertensive drug treatment alone, and a combination intervention that included both salt substitution and antihypertensive drug treatment. We evaluated the cost-effectiveness of each intervention option for the overall prehypertensive population, prehypertensive individuals at high risk of CVD outcomes (with a 10-year risk of CVD ≥10%), and across age groups (starting at ages 40, 50, 60, and 70 years). The 10-year risk of CVD for prehypertensive individuals was assessed using the risk prediction model developed in the CKB cohort (CKB-CVD model).^32^

### Model Parameters

#### Transition Probabilities

Using CKB data, we calculated the transition probabilities from prehypertensive to various disease states and death for the overall prehypertensive population (N=151 788) and individuals at high risk of CVD (N=54 926). These probabilities were computed for different age groups (35–44, 45–54, 55–64, and 65–75 years) to determine the 10-year transition probabilities, which were then treated as the average transition probabilities for each age group, demonstrating how transition probabilities increase with age (Table S1). The 10-year transition probability was converted to a 1-year transition probability using the following formula: r=1−exp(ln(1−p)/10), where r represents the 1-year incidence rate and p represents the cumulative incidence over 10 years.

For transitions between different CVD events, due to the limited number of study participants, the average transition probabilities for age groups 35 to 75, 45 to 75, 55 to 75, and 65 to 75 years were used to approximate the average transition probabilities for starting ages of 40, 50, 60, and 70 years (Table S2).

#### Intervention Effectiveness

A study in the Chinese population showed that salt substitution intervention in individuals with normal blood pressure could reduce SBP by ≈2 mm Hg after 2 years of follow-up.^18^ Given previous studies demonstrating a log-linear relationship between blood pressure reduction and decreased risk of CVD,^33^ we used data from the CKB to calculate the association between a 10 mm Hg reduction in blood pressure and various CKB events in different age groups, which was then linearly translated to the effects of a 2 mm Hg reduction in blood pressure on CVD events. We calculated regression dilution ratios using Rosner regression method to adjust for regression dilution bias.^34^ The detailed calculation process is shown in the Supplemental Methods.

To allow comparability between the effects of salt substitution intervention and antihypertensive drug treatment, we leveraged data on the impact of antihypertensive treatment on the blood pressure in prehypertensive populations from the multicenter, multiethnic Heart Outcomes Prevention Evaluation (HOPE)-3 study, which was comprised of 29% Chinese participants.^17^ The log-linear relationship between blood pressure and CVD was then used to calculate the impact of antihypertensive drug treatment on CVD events. The HOPE-3 trial found that antihypertensive treatment could reduce SBP by ≈5.6 mm Hg over a follow-up of 5.6 years for those with baseline blood pressure levels ranging from 131.6 to 143.5 mm Hg.

The combined effect of salt substitution and antihypertensive drug treatments was calculated by multiplying the effect values of the 2 interventions.

### Costs and Utilities

As this study was conducted from a societal perspective, both direct and indirect costs were considered. Direct costs consisted of hospitalization and chronic stage costs of CVD events for all groups, as well as additional intervention costs for intervention groups.

We obtained the cost of antihypertensive drug treatment from a previous cost-effectiveness study of hypertension treatment in China,^20^ where the cost was determined by combining the proportion of antihypertensive drug use with the cost of antihypertensive drugs from a cross-sectional survey in China.^35^ Since the implementation of centralized drug procurement policy in China in 2018, which significantly reduced the prices of selected drugs, we utilized data from a cross-sectional study conducted in hospitals in China to obtain the extent of price reductions for selected antihypertensive drugs, thereby estimating the current annual cost of antihypertensive drug treatment.^36^ The cost of salt substitution interventions was obtained from a cluster RCT of a household salt substitution intervention conducted in China, mainly including the additional costs of substitute salt and promotional materials.^20^ The study calculated the annual cost per capita difference due to salt substitution by comparing the price per unit of substitute salt to regular salt and assuming an average salt consumption of 10 g/d per prehypertensive person, which is lower than that of per hypertensive person.^37,38^ Hospitalization costs were sourced from the China Health Statistical Yearbook 2020.^22^ Costs associated with CVD treatment during the chronic stage were determined from published studies on the Chinese population.^23^

Indirect costs, such as productivity losses due to disease, disability, or death, were estimated using the human capital approach. The national average salary from the labor market in 2019 was used to quantify productivity losses caused by hospitalization or premature death of patients and caregiving by 1 family member, assuming that all lost time would have been used for production. Relevant data were sourced from the China Health Statistics Yearbook and the China Statistical Yearbook.^22,24^

The utility values of each health state were estimated to calculate the QALYs. The utility of a disease-free state without treatment was set to 1, and the utility of death was 0. Other utility values were derived from published cost-effectiveness studies and published surveys of the Chinese population.^25,26^

### Sensitivity Analyses

Several sensitivity analyses were performed to assess the model’s robustness and identify potential sources of uncertainty. One-way sensitivity analyses were performed on model parameters using the plausibility ranges detailed in Table 1. Additionally, probabilistic sensitivity analysis was performed using Monte Carlo simulations (N=5000 iterations) to explore the impact of simultaneous changes in multiple parameters. The uncertainties were depicted using tornado diagrams and cost-effectiveness acceptability curves, respectively.

We also conducted the following sensitivity analyses to assess the robustness of the results: (1) we adjusted the range of prehypertension to 120–139/80–89 mm Hg and the threshold for high CVD risk to 20%. Transition probabilities have been calculated for the corresponding blood pressure or CVD risk range population based on the CKB study. (2) We used hazard ratio associated with a 5-mm Hg reduction in SBP due to blood pressure-lowering medications for CVD events (hazard ratio, 0.89 [95% CI, 0.81–0.97]). The ratio was obtained from a meta-analysis of individual participant-level RCTs in prehypertensive individuals with SBP of 130 to 139 mm Hg.^10^ (3) We also considered adherence to drug treatment. We set a medication adherence rate of 75% for the first 5 years and 50% after 5 years according to the HOPE-3 trial, where 75% of the participants were still taking medications at 5 years.^17^

### Results

#### Interventions in All Prehypertensive Individuals

**Figure 1.**
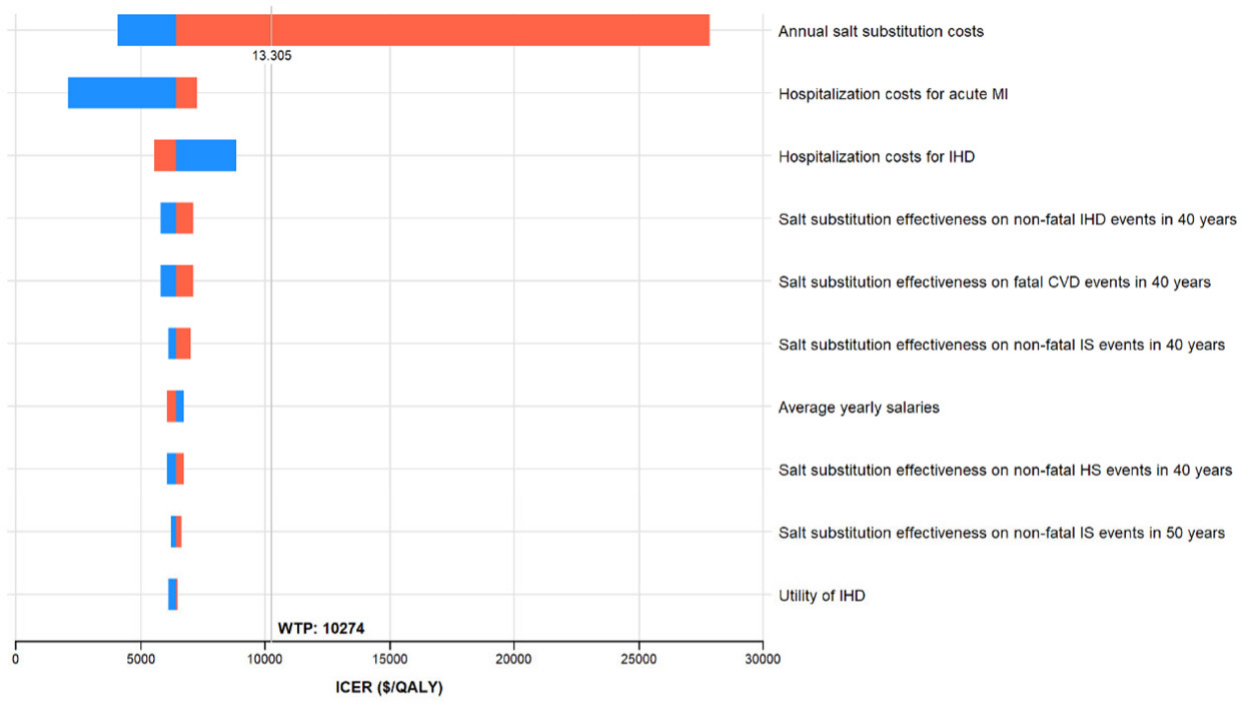
**One-way sensitivity analyses for salt substitution intervention vs no intervention from age 40 years in prehypertensive individuals.** This figure only shows the top 10 variables that had the greatest influence on the incremental cost-effectiveness ratio (ICER). The blue and red bars represent parameter ranges that are lower or higher than the base-case values shown in Table 1, respectively. The willing to pay (WTP) line represents gross domestic product per capita in 2019. CVD indicates cardiovascular disease; HS, hemorrhagic stroke; IHD, ischemic heart disease; IS, ischemic stroke; MI, myocardial infarction; and QALY, quality-adjusted life year.

**Figure 2.**
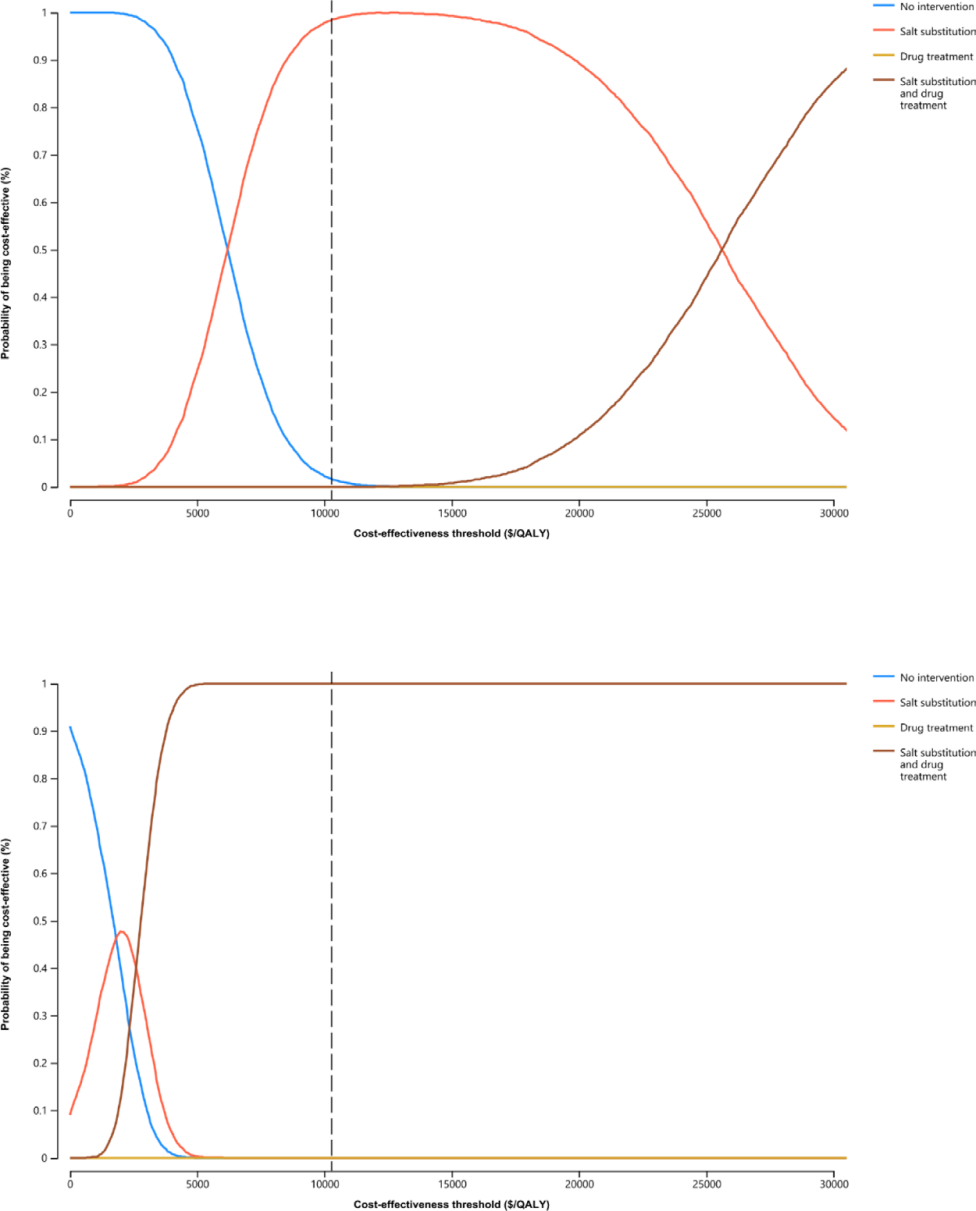
**Cost-effectiveness acceptability curves for intervention strategies in prehypertensive individuals from 40 years. Top**, All individuals. **Bottom**, High cardiovascular disease risk individuals. The dashed lines represent the gross domestic product per capita in 2019. QALY indicates quality-adjusted life year.

At a willingness-to-pay threshold of the GDP per capita, salt substitution intervention from the age of 40 was cost-effective compared with no intervention in prehypertensive individuals, whereas antihypertensive drug treatment was not cost-effective compared with no intervention, with an ICER of $6413.62/QALY and $27 738.80/QALY (extended dominance), respectively (Table 2). The combination intervention is the most effective strategy, yielding the highest lifetime QALYs of 16.41, with an additional 0.08 QALYs gained per person compared with no intervention. This result remained robust in sensitivity analyses. One-way sensitivity analysis revealed that the cost of salt substitution had the most significant impact on the cost-effectiveness of the intervention, with all other parameters within their ranges falling inside the willingness-to-pay threshold (Figure 1). The cost of substituting salt at the threshold of the GDP per capita was approximately $13.3/y. Probabilistic sensitivity analysis indicated that, at the threshold of the GDP per capita, there was a 98.3% probability that the salt substitution intervention was cost-effective (Figure 2A). Furthermore, salt substitution intervention was cost-effective for individuals with blood pressure in the range of 120–139/80–89 mm Hg, with an ICER of $6849.26/QALY compared with no intervention (Table S3).

**Table 2.**
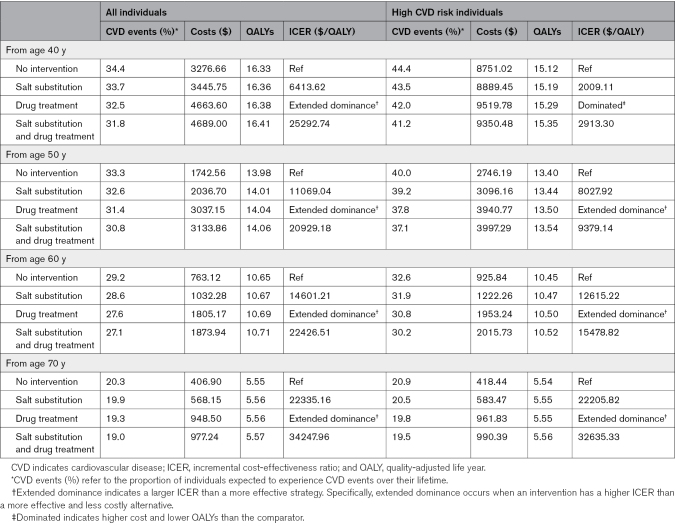
Cost-Effectiveness of Intervention Strategies in Prehypertensive Individuals by Age

#### Interventions in High CVD Risk Prehypertensive Individuals

When compared with a no-intervention scenario, interventions involving salt substitution, antihypertensive drug treatment, and a combination of the 2 were cost-effective among high CVD risk prehypertensive individuals, with ICERs of $2009.11/QALY, $4573.81/QALY, and $2639.05/QALY, respectively. Among these, the combination intervention of salt substitution and antihypertensive drug treatment from individuals aged 40 years with high CVD risk in the prehypertensive population was identified as the most cost-effective strategy, with an ICER of $2913.30/QALY compared with salt substitution intervention alone (Table 2).

**Figure 3.**
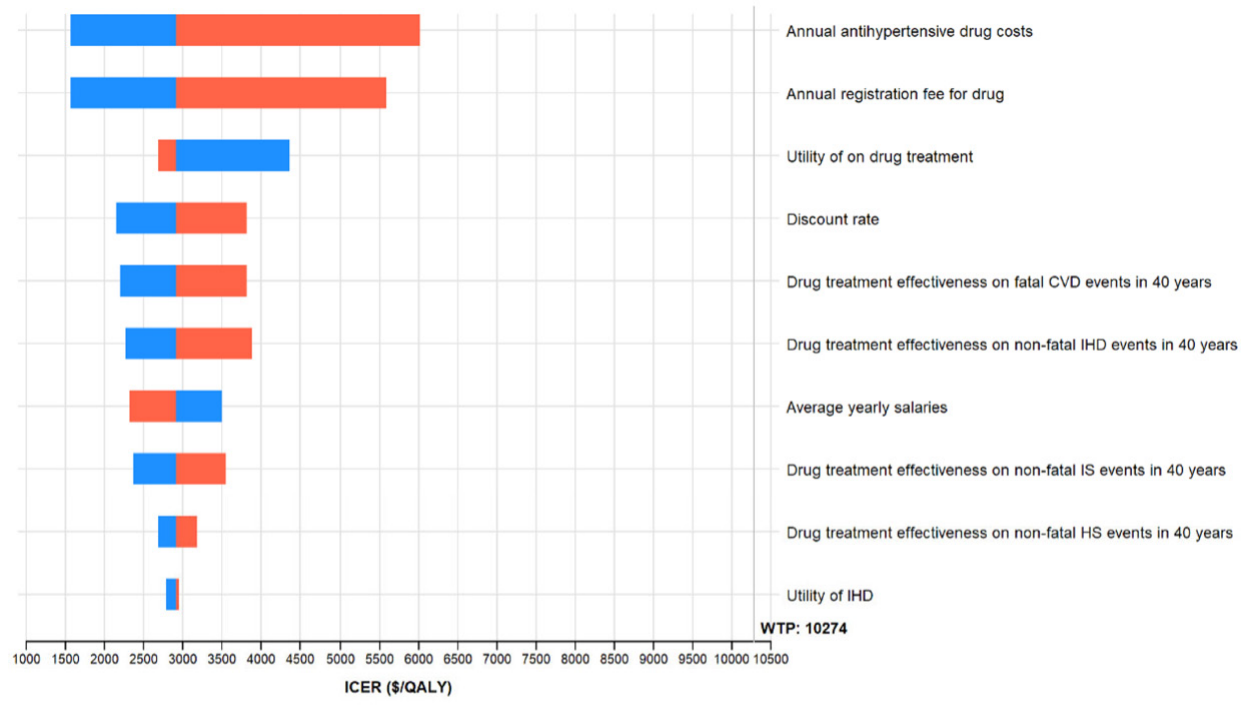
**One-way sensitivity analyses for salt substitution and drug treatment intervention vs salt substitution intervention from age 40 years in prehypertensive individuals with high cardiovascular disease (CVD) risk.** This figure only shows the top 10 variables that had the greatest influence on the incremental cost-effectiveness ratio (ICER). The blue and red bars represent parameter ranges that are lower or higher than the base-case values shown in Table 1, respectively. The willing to pay (WTP) line indicates the gross domestic product per capita in 2019. HS indicates hemorrhagic stroke; IHD, ischemic heart disease; IS, ischemic stroke; MI, myocardial infarction; and QALY, quality-adjusted life year.

This finding remained robust across various sensitivity analyses. One-way sensitivity analysis revealed that the ICERs remained within the willingness-to-pay threshold across all parameter ranges, with the cost associated with antihypertensive drug treatment having the most significant impact on the results (Figure 3). Probabilistic sensitivity analysis indicated that the combination intervention had a 99.9% probability of being cost-effective at the threshold of the GDP per capita (Figure 2B).

Sensitivity analyses for populations with blood pressure of 120–139/80–89 mm Hg and higher CVD risk showed that the ICERs for the combination intervention ($1673.53/QALY and $519.24/QALY) were slightly lower than the base-case analysis (Tables S3 and S4), indicating that the combination intervention was a more favorable strategy. In sensitivity analyses using the effect of antihypertensive drug treatment on CVD from previous RCT meta-analyses and considering noncompliance rates, the ICERs for the combination intervention ($4019.44/QALY and $7843.77/QALY) were slightly higher than the base-case value but remained within the range of the GDP per capita (Tables S5 and S6).

#### Interventions Starting at Different Ages

The findings indicated that interventions starting at a younger population resulted in a higher reduction in the proportion of CVD events, with lower ICERs for each intervention strategy. For example, among populations at high risk of CVD events in the prehypertensive stage, a combination intervention beginning at age 40 years, compared with a salt substitution intervention alone, reduced CVD events by ≈5.3%, with an ICER of $2913.30/QALY. For the group beginning at 70 years, the reduction was about 4.9%, with an ICER of $32 635.33/QALY (Table 2).

### Discussion

This study found that salt substitution intervention was cost-effective for the entire prehypertensive population. However, neither antihypertensive treatment nor a combination of the salt substitution intervention and antihypertensive treatment proved cost-effective among the prehypertensive population aged >40 years. The price of salt substitution had a significant impact on the results. For prehypertensive individuals who are at high risk of CVD, salt substitution intervention plus antihypertensive treatment was the most cost-effective. Furthermore, interventions were more cost-effective when starting at an earlier age.

In this study, the effect of antihypertensive treatment was not measured as a direct reduction in the risk of cardiovascular events. Instead, it was measured as a reduction in blood pressure associated with CVD events, which was then calculated as a reduction in CVD events based on the association between blood pressure and CVD events observed in the CKB study. The main reason for this is that the results can be compared with those of the salt substitution intervention. Currently, there is a lack of evidence on the efficacy of salt substitution intervention on CVD events in the prehypertensive population, but its effect on blood pressure has been studied. In addition, previous studies have shown that associations between blood pressure and vascular diseases were stronger in younger age groups, and blood pressure was more strongly associated with hemorrhagic stroke than with other vascular diseases in the Chinese population.^33,39^ Therefore, we estimated associations for different age groups and outcomes using data from CKB to better reflect the influence of age and outcomes. The results remained robust in the sensitivity analyses, which used the effect of antihypertensive treatment on CVD events directly.

Two RCTs were conducted in China to investigate the effects and cost-effectiveness of salt substitution. The findings of these trials demonstrate that salt substitution is an effective and cost-saving method for reducing the risk of hypertension and CVD events. However, it is crucial to highlight that there are differences between the populations in the above studies and those in this study. The Salt Substitute and Stroke Study was a 5-year RCT conducted among participants living in rural China who had a history of stroke or were >60 years old with uncontrolled high blood pressure.^38^ The DECIDE-Salt study (Diet, Exercise and Cardiovascular Health—Salt Reduction Strategies for the Elderly in Nursing Homes in China) was conducted among older adults with normal blood pressure in elderly care facilities in China for 2 years.^11^ Both within-trial economic evaluations showed that salt substitution was a more cost-saving intervention than regular salt.^20,40^ Our model-based study showed that salt substitution is a cost-effective, although not cost-saving, strategy for prehypertensive individuals. The lower effect of salt substitution and lower CVD risk in the prehypertensive population compared with uncontrolled high blood pressure and older populations may be the main reasons.

Three cost-utility analyses have explored the cost-effectiveness of antihypertensive drug treatment for Chinese prehypertension individuals. However, the findings of these studies were inconsistent. Chen et al^14^ found that drug treatment would reduce the incidence of hypertension over a lifetime in Chinese adults with prehypertension. However, they also found that there was only a 10% probability that drug treatment would be cost-effective at a willingness-to-pay threshold of GDP per capita in 2014. Zhou et al^15^ found that drug treatment was not cost-effective for prehypertensive individuals aged ≥65 years without CVD, compared with nondrug treatment. In contrast, Li et al^16^ found that antihypertensive treatment for prehypertensive adults with high CVD risk was cost-effective under the threshold of GDP per capita in 2022 (Int$ 21318), with an ICER of Int$ 13321.29 per QALY over a 10-year time horizon. Apart from the differences in the variables of transition probabilities and antihypertensive medication effect, the main explanation for the different conclusions could be the differences in the study population. Our study showed that antihypertensive treatment was also not cost-effective in the overall prehypertensive population compared with no treatment ($27 738.80/QALY), but it was cost-effective in prehypertensive populations with a high risk of CVD ($4573.81/QALY).

It was found that the interventions were more cost-effective when starting at a younger age. Previous studies have shown that the associations between blood pressure and CVD events were stronger in younger than in older individuals.^33^ The Mendelian randomization study based on CVD found that the hazard ratio for major vascular events per 10 mm Hg higher genetically predicted SBP was twice as high in younger than in older individuals, suggesting that younger individuals could benefit more from blood pressure-lowering.^39^ Li et al^16^ found that initiating antihypertensive drug treatment earlier could prevent more CVD cases and gain more incremental QALYs. This emphasizes the importance of early intervention for younger people.

To our knowledge, this is the first study to investigate the costs and effectiveness of salt substitution intervention and a combination intervention of antihypertensive drug treatment and salt substitution in prehypertensive individuals, as well as to compare the cost-effectiveness of antihypertensive drug treatment with salt substitution intervention. To ensure the homogeneity of data, the CKB study was used to calculate transition probabilities and associations between blood pressure reduction and CVD outcomes. Although the CKB population was not designed to be representative of the general prehypertensive population in China, the inclusion of a large number of participants from diverse regions and population characteristics helps to provide important evidence that can be generalized to the broader prehypertensive population.

There were some limitations to this study. Due to the lack of evidence on the direct effect of salt substitution on CVD events, we assumed that the reduction in blood pressure would decrease the risk of CVD events. Future RCTs on salt substitution intervention in prehypertensive individuals are needed. Adverse clinical events such as hypotension, hyperkalemia, and hyponatremia were not accounted for in this study due to a lack of relevant evidence. The study only considered household-level salt substitution interventions, not community- or government-level interventions, which would probably produce more health benefits with lower costs. The variables in this study were mostly from studies in China, so generalizing the findings to other countries should be done with caution.

### Perspectives

In summary, this study has shown that substituting household salt is a cost-effective intervention for all prehypertensive individuals in the context of an economic level like China. Antihypertensive drug treatment, on the contrary, is only cost-effective for prehypertensive individuals at high risk of CVD. These findings provide further evidence for the primary prevention and control of hypertension and CVD in China.

### ARTICLE INFORMATION

#### Acknowledgments

The most important acknowledgment is to the participants in the study and the members of the survey teams in each of the 10 regional centers, as well as to the project development and management teams based at Beijing, Oxford, and the 10 regional centers.

#### Sources of Funding

This study was supported by National Natural Science Foundation of China (82192904, 82388102, and 82192900) and grants (2023YFC2509400) from the National Key R&D Program of China. The China Kadoorie Biobank baseline survey and the first re-survey were supported by a grant from the Kadoorie Charitable Foundation in Hong Kong. The long-term follow-up is supported by grants from the UK Wellcome Trust (212946/Z/18/Z, 202922/Z/16/Z, 104085/Z/14/Z, and 088158/Z/09/Z), grants (2016YFC0900500) from the National Key R&D Program of China, National Natural Science Foundation of China (81390540, 91846303, and 81941018), and Chinese Ministry of Science and Technology (2011BAI09B01). The funders had no role in the study design, data collection, data analysis and interpretation, writing of the report, or the decision to submit the article for publication.

#### Disclosures

None.

#### Supplemental Material

Supplemental Methods

Tables S1–S6

Figure S1

## Supplementary Material


