# Supplementary material for: Cost-Effectiveness of Salt Substitution and Antihypertensive Drug Treatment in Chinese Prehypertensive Adults
Source: Hypertension. 2024 Oct 28;81(12):2529–39. doi: 10.1161/HYPERTENSIONAHA.124.23412 (PMC11578052; doi:10.1161/HYPERTENSIONAHA.124.23412)
Supplement: Supplementary file 1 [file hyp-81-2529-s001.docx]

**Cost-Effectiveness of Salt Substitution and Antihypertensive Drug Treatment in Chinese Prehypertensive Adults**

Zhijia Sun, Haijun Zhang, Yinqi Ding, Canqing Yu, Dianjianyi Sun, Yuanjie Pang, Pei Pei, Ling Yang, Yiping Chen, Huaidong Du, Weijie Hu, Daniel Avery, Junshi Chen, Zhengming Chen, Liming Li, Jun Lv*, on behalf of the China Kadoorie Biobank Collaborative Group

**Supplementary Online Content**

[**Members of the China Kadoorie Biobank collaborative group 2**](#_Toc178928180)

[**Supplemental Methods 3**](#_Toc178928181)

[**References 4**](#_Toc178928182)

[Table S1. Disease progression inputs for prehypertensive individuals 5](#_Toc178928183)

[Table S2. Disease progression inputs in CVD individuals 6](#_Toc178928184)

[Table S3. Sensitivity analysis of the cost-effectiveness of intervention strategies in prehypertensive individuals (120mmHg≤SBP<140mmHg) 7](#_Toc178928185)

[Table S4. Sensitivity analysis of the cost-effectiveness of intervention strategies in individuals with a 10-year CVD risk of more than 20% 8](#_Toc178928186)

[Table S5. Sensitivity analysis of the cost-effectiveness of intervention strategies based on drug treatment effectiveness from RCT studies 9](#_Toc178928187)

[Table S6. Sensitivity analysis of the cost-effectiveness of intervention strategies based on low compliance rate 11](#_Toc178928188)

[Figure S1. Structure of the Markov model 12](#_Toc178928189)

**Members of the China Kadoorie Biobank collaborative group**

**International Steering Committee:** Junshi Chen, Zhengming Chen (PI), Robert Clarke, Rory Collins, Liming Li (PI), Jun Lv, Richard Peto, Robin Walters.

**International Co-ordinating Centre, Oxford:** Daniel Avery, Maxim Barnard, Derrick Bennett, Lazaros Belbasis, Ruth Boxall, Ka Hung Chan, Yiping Chen, Zhengming Chen, Charlotte Clarke, Johnathan Clarke; Robert Clarke, Huaidong Du, Ahmed Edris Mohamed, Hannah Fry, Simon Gilbert, Pek Kei Im, Andri Iona, Maria Kakkoura, Christiana Kartsonaki, Hubert Lam, Kuang Lin, James Liu, Mohsen Mazidi, Iona Millwood, Sam Morris, Qunhua Nie, Alfred Pozarickij, Maryanm Rahmati, Paul Ryder, Saredo Said, Dan Schmidt, Becky Stevens, Iain Turnbull, Robin Walters, Baihan Wang, Lin Wang, Neil Wright, Ling Yang, Xiaoming Yang, Pang Yao.

**National Co-ordinating Centre, Beijing:** Xiao Han, Can Hou, Qingmei Xia, Chao Liu, Jun Lv, Pei Pei, Dianjianyi Sun, Canqing Yu, Lang Pan.

**10 Regional Co-ordinating Centres:**

**Qingdao CDC:** Zengchang Pang, Ruqin Gao, Shanpeng Li, Haiping Duan, Shaojie Wang, Yongmei Liu, Ranran Du, Yajing Zang, Liang Cheng, Xiaocao Tian, Hua Zhang, Yaoming Zhai, Feng Ning, Xiaohui Sun, Feifei Li. **Licang CDC:** Silu Lv, Junzheng Wang, Wei Hou. **Heilongjiang Provincial CDC:** Wei Sun, Shichun Yan, Xiaoming Cui. **Nangang CDC:** Chi Wang, Zhenyuan Wu,Yanjie Li, Quan Kang. **Hainan Provincial CDC:** Huiming Luo, Tingting Ou. **Meilan CDC:** Xiangyang Zheng, Zhendong Guo, Shukuan Wu, Yilei Li, Huimei Li. **Jiangsu Provincial CDC:** Ming Wu, Yonglin Zhou, Jinyi Zhou, Ran Tao, Jie Yang, Jian Su. **Suzhou CDC:** Fang Liu, Jun Zhang, Yihe Hu, Yan Lu, Liangcai Ma, Aiyu Tang, Shuo Zhang, Jianrong Jin, Jingchao Liu. **Guangxi Provincial CDC:** Mei Lin, Zhenzhen Lu. **Liuzhou CDC:** Lifang Zhou, Changping Xie, Jian Lan,Tingping Zhu,Yun Liu, Liuping Wei, Liyuan Zhou, Ningyu Chen, Yulu Qin, Sisi Wang. **Sichuan Provincial CDC:** Xianping Wu, Ningmei Zhang, Xiaofang Chen, Xiaoyu Chang. **Pengzhou CDC:** Mingqiang Yuan, Xia Wu, Xiaofang Chen, Wei Jiang, Jiaqiu Liu, Qiang Sun. **Gansu Provincial CDC:** Faqing Chen, Xiaolan Ren, Caixia Dong. **Maiji CDC:** Hui Zhang, Enke Mao, Xiaoping Wang, Tao Wang, Xi zhang. **Henan Provincial CDC:** Kai Kang, Shixian Feng, Huizi Tian, Lei Fan. **Huixian CDC:** XiaoLin Li, Huarong Sun, Pan He, Xukui Zhang. **Zhejiang Provincial CDC:** Min Yu, Ruying Hu, Hao Wang. **Tongxiang CDC**: Xiaoyi Zhang, Yuan Cao, Kaixu Xie, Lingli Chen, Dun Shen. **Hunan Provincial CDC:** Xiaojun Li, Donghui Jin, Li Yin, Huilin Liu, Zhongxi Fu. **Liuyang CDC:** Xin Xu, Hao Zhang, Jianwei Chen,Yuan Peng, Libo Zhang, Chan Qu.

**Supplemental Methods**

We excluded participants who had a self-reported history of cardiovascular diseases (CVD, including myocardial infarction, stroke, or transient ischemic attack), hypertension, or were taking blood-pressure-lowering medication at baseline of the China Kadoorie Biobank (CKB) study. The remaining participants (N=151,788) in the analysis ranged in age from 35 to 75 years old and had blood pressure levels of 130-139/80-89 mmHg.

We used the Cox proportional hazards model to calculate hazard ratios (HRs) and 95% confidence intervals (CIs) for the associations between a 10 mmHg reduction in systolic blood pressure (SBP) and the first occurrence of non-fatal ischemic heart disease (IHD), non-fatal ischemic stroke (IS), non-fatal hemorrhagic stroke (HS), and fatal CVD events. Models were stratified by study area and adjusted for sex, education, area, smoking, alcohol intake, and body mass index (BMI). We calculated the HRs for four age groups separately (35-44, 45-54, 55-64, and 65-75 years).

To adjust for regression dilution bias, we generated regression dilution ratios (RDR) using repeated SBP measurements taken at baseline and the first resurvey of the CKB study. We used Rosner’s regression method to calculate RDR^1^, which is the slope of the regression line between baseline and resurvey SBP values, and further calculated the corrected HRs. Since SBP was positively and log-linearly associated with CVD events, we further transferred the HRs for a 10 mmHg SBP reduction to 5.6 mmHg and 2 mmHg reduction separately.

# References

1. Rosner B, Willett WC, Spiegelman D. Correction of logistic regression relative risk estimates and confidence intervals for systematic within-person measurement error. *Stat Med*. 1989;8:1051-1069; discussion 1071-1053. doi: 10.1002/sim.4780080905

2. Blood Pressure Lowering Treatment Trialists Collaboration. Pharmacological blood pressure lowering for primary and secondary prevention of cardiovascular disease across different levels of blood pressure: an individual participant-level data meta-analysis. *Lancet*. 2021;397:1625-1636. doi: 10.1016/S0140-6736(21)00590-0

# Table S1. Disease progression inputs for prehypertensive individuals

| Transition | 35-44y | 45-54y | 55-64y | 65-75y |
| --- | --- | --- | --- | --- |
| All individuals |  |  |  |  |
| From prehypertension to |  |  |  |  |
| IS | 0.0158 | 0.0445 | 0.0794 | 0.1372 |
| HS | 0.0030 | 0.0058 | 0.0102 | 0.0169 |
| Acute MI | 0.0012 | 0.0029 | 0.0048 | 0.0087 |
| Other IHD | 0.0188 | 0.0459 | 0.0718 | 0.1166 |
| CVD death | 0.0028 | 0.0082 | 0.0209 | 0.0814 |
| Death from other causes | 0.0146 | 0.0319 | 0.0673 | 0.1713 |
| Individuals at high risk for CVD |  |  |  |  |
| From prehypertension to |  |  |  |  |
| IS | 0.0940 | 0.1039 | 0.1113 | 0.1407 |
| HS | 0.0121 | 0.0104 | 0.0135 | 0.0174 |
| Acute MI | 0.0054 | 0.0060 | 0.0063 | 0.0091 |
| Other IHD | 0.1020 | 0.0945 | 0.0913 | 0.1204 |
| CVD death | 0.0128 | 0.0175 | 0.0304 | 0.0841 |
| Death from other causes | 0.0262 | 0.0418 | 0.0754 | 0.1735 |

IS: ischemic stroke; HS: hemorrhagic stroke; MI: myocardial infarction; IHD: ischemic heart disease; CVD: cardiovascular disease.

# Table S2. Disease progression inputs in CVD individuals

| Transition | 35-75y | 45-75y | 55-75y | 65-75y |
| --- | --- | --- | --- | --- |
| From IS to |  |  |  |  |
| HS | 0.0047 | 0.0048 | 0.0049 | 0.0054 |
| Acute MI | 0.0022 | 0.0023 | 0.0026 | 0.0032 |
| Other IHD | 0.0283 | 0.0295 | 0.0322 | 0.0362 |
| CVD death | 0.0172 | 0.0184 | 0.0233 | 0.0331 |
| Death from other causes | 0.0228 | 0.0245 | 0.0312 | 0.0443 |
| From HS to |  |  |  |  |
| IS | 0.0542 | 0.0581 | 0.0620 | 0.0616 |
| Acute MI | 0.0010 | 0.0011 | 0.0010 | 0.0010 |
| Other IHD | 0.0129 | 0.0145 | 0.0179 | 0.0203 |
| CVD death | 0.0666 | 0.0724 | 0.0923 | 0.1206 |
| Death from other causes | 0.0370 | 0.0411 | 0.0550 | 0.0777 |
| From Acute MI to |  |  |  |  |
| IS | 0.0286 | 0.0305 | 0.0351 | 0.0505 |
| HS | 0.0015 | 0.0017 | 0.0017 | 0.0016 |
| Other IHD | 0.1436 | 0.1486 | 0.1560 | 0.1788 |
| CVD death | 0.0353 | 0.0380 | 0.0465 | 0.0655 |
| Death from other causes | 0.0211 | 0.0229 | 0.0279 | 0.0406 |
| From other IHD to |  |  |  |  |
| IS | 0.0318 | 0.0344 | 0.0382 | 0.0438 |
| HS | 0.0023 | 0.0024 | 0.0027 | 0.0032 |
| Acute MI | 0.0044 | 0.0047 | 0.0054 | 0.0062 |
| CVD death | 0.0149 | 0.0164 | 0.0218 | 0.0321 |
| Death from other causes | 0.0189 | 0.0208 | 0.0275 | 0.0406 |

IS: ischemic stroke; HS: hemorrhagic stroke; MI: myocardial infarction; IHD: ischemic heart disease; CVD: cardiovascular disease.

# Table S3. Sensitivity analysis of the cost-effectiveness of intervention strategies in prehypertensive individuals (120mmHg≤SBP<140mmHg)

|  | All individuals | | |  | High CVD risk individuals | | |
| --- | --- | --- | --- | --- | --- | --- | --- |
|  | Costs ($) | QALYs | ICER ($/QALY) |  | Costs ($) | QALYs | ICER ($/QALY) |
| From age 40 years |  |  |  |  |  |  |  |
| No intervention | 3088.30 | 16.39 | Ref |  | 8798.27 | 15.11 | Ref |
| Salt substitution | 3256.88 | 16.42 | 6849.26 |  | 8920.25 | 15.19 | Extended dominance^a^ |
| Drug treatment | 4471.84 | 16.45 | Extended dominance^a^ |  | 9403.03 | 15.32 | Dominated^b^ |
| Salt substitution and drug treatment | 4504.45 | 16.47 | 24799.47 |  | 9222.26 | 15.38 | 1673.53 |
| From age 50 years |  |  |  |  |  |  |  |
| No intervention | 1655.47 | 14.04 | Ref |  | 2730.42 | 13.42 | Ref |
| Salt substitution | 1947.19 | 14.07 | 11636.60 |  | 3082.49 | 13.46 | 7992.71 |
| Drug treatment | 2950.11 | 14.10 | Extended dominance^a^ |  | 3910.67 | 13.53 | Extended dominance^a^ |
| Salt substitution and drug treatment | 3050.62 | 14.12 | 21004.83 |  | 3967.81 | 13.57 | 8567.84 |
| From age 60 years |  |  |  |  |  |  |  |
| No intervention | 733.51 | 10.69 | Ref |  | 902.89 | 10.49 | Ref |
| Salt substitution | 1001.91 | 10.71 | 15768.74 |  | 1200.39 | 10.51 | 13411.36 |
| Drug treatment | 1776.51 | 10.73 | Extended dominance^a^ |  | 1931.14 | 10.55 | Extended dominance^a^ |
| Salt substitution and drug treatment | 1846.76 | 10.75 | 23066.36 |  | 1994.94 | 10.57 | 15574.57 |
| From age 70 years |  |  |  |  |  |  |  |
| No intervention | 394.89 | 5.57 | Ref |  | 409.04 | 5.56 | Ref |
| Salt substitution | 556.54 | 5.58 | 24650.17 |  | 575.57 | 5.56 | 24503.87 |
| Drug treatment | 934.84 | 5.58 | Extended dominance^a^ |  | 951.28 | 5.57 | Extended dominance^a^ |
| Salt substitution and drug treatment | 963.98 | 5.59 | 32748.16 |  | 980.20 | 5.58 | 30882.11 |

SBP: systolic blood pressure; CVD: cardiovascular disease; $: Chinese Yuan; QALYs: quality-adjusted life year; ICER: incremental cost-effectiveness ratio.

^a^ Extended dominance indicates larger ICER than a more effective strategy. ^b^ Dominated indicates higher cost and lower QALYs than the comparator.

# Table S4. Sensitivity analysis of the cost-effectiveness of intervention strategies in individuals with a 10-year CVD risk of more than 20%

|  | Costs ($) | QALYs | ICER ($/QALY) |
| --- | --- | --- | --- |
| From age 40 years |  |  |  |
| No intervention | 12486.56 | 14.27 | Ref |
| Salt substitution | 12646.49 | 14.36 | Dominated^a^ |
| Drug treatment | 12916.10 | 14.49 | Dominated^a^ |
| Salt substitution and drug treatment | 12639.95 | 14.56 | 519.24 |
| From age 50 years |  |  |  |
| No intervention | 4113.63 | 12.66 | Ref |
| Salt substitution | 4562.30 | 12.72 | Extended dominance^b^ |
| Drug treatment | 5197.33 | 12.81 | Extended dominance^b^ |
| Salt substitution and drug treatment | 5201.71 | 12.87 | 5332.43 |
| From age 60 years |  |  |  |
| No intervention | 1246.88 | 10.11 | Ref |
| Salt substitution | 1604.02 | 10.14 | Extended dominance^b^ |
| Drug treatment | 2258.13 | 10.19 | Extended dominance^b^ |
| Salt substitution and drug treatment | 2309.39 | 10.22 | 10282.76 |
| From age 70 years |  |  |  |
| No intervention | 479.63 | 5.48 | Ref |
| Salt substitution | 660.30 | 5.49 | 20173.10 |
| Drug treatment | 1027.61 | 5.50 | Extended dominance^b^ |
| Salt substitution and drug treatment | 1055.16 | 5.51 | 24448.68 |

CVD: cardiovascular disease; QALYs: quality-adjusted life year; ICER: incremental cost-effectiveness ratio.

^a^ Dominated indicates higher cost and lower QALYs than the comparator.

^b^ Extended dominance indicates larger ICER than a more effective strategy.

# Table S5. Sensitivity analysis of the cost-effectiveness of intervention strategies based on drug treatment effectiveness from RCT studies

|  | All individuals | | |  | High CVD risk individuals | | |
| --- | --- | --- | --- | --- | --- | --- | --- |
|  | Costs ($) | QALYs | ICER ($/QALY) |  | Costs ($) | QALYs | ICER ($/QALY) |
| From age 40 years |  |  |  |  |  |  |  |
| No intervention | 3276.66 | 16.33 | Ref |  | 8751.02 | 15.12 | Ref |
| Salt substitution | 3445.75 | 16.36 | 6413.62 |  | 8889.45 | 15.19 | 2009.11 |
| Drug treatment | 4669.82 | 16.38 | Extended dominance^a^ |  | 9631.89 | 15.27 | Dominated^b^ |
| Salt substitution and drug treatment | 4690.65 | 16.40 | 26979.82 |  | 9443.49 | 15.33 | 4019.44 |
| From age 50 years |  |  |  |  |  |  |  |
| No intervention | 1742.56 | 13.98 | Ref |  | 2746.19 | 13.40 | Ref |
| Salt substitution | 2036.70 | 14.01 | 11069.04 |  | 3096.16 | 13.44 | 8027.92 |
| Drug treatment | 2993.32 | 14.04 | Extended dominance^a^ |  | 3888.95 | 13.50 | Extended dominance^a^ |
| Salt substitution and drug treatment | 3089.95 | 14.07 | 18628.24 |  | 3944.46 | 13.54 | 8567.84 |
| From age 60 years |  |  |  |  |  |  |  |
| No intervention | 763.12 | 10.65 | Ref |  | 925.84 | 10.45 | Ref |
| Salt substitution | 1032.28 | 10.67 | 14601.21 |  | 1222.26 | 10.47 | Extended dominance^a^ |
| Drug treatment | 1750.52 | 10.70 | Extended dominance^a^ |  | 1891.46 | 10.51 | Extended dominance^a^ |
| Salt substitution and drug treatment | 1820.10 | 10.72 | 16893.40 |  | 1954.82 | 10.53 | 12226.35 |
| From age 70 years |  |  |  |  |  |  |  |
| No intervention | 406.90 | 5.55 | Ref |  | 418.44 | 5.54 | Ref |
| Salt substitution | 568.15 | 5.56 | Extended dominance^a^ |  | 583.47 | 5.55 | Extended dominance^a^ |
| Drug treatment | 904.06 | 5.57 | Extended dominance^a^ |  | 916.14 | 5.56 | Extended dominance^a^ |
| Salt substitution and drug treatment | 933.21 | 5.57 | 20156.89 |  | 945.12 | 5.57 | 19496.39 |

SBP: systolic blood pressure; CVD: cardiovascular disease; $: Chinese Yuan; QALYs: quality-adjusted life year; ICER: incremental cost-effectiveness ratio.

The association between SBP reduction and major cardiovascular events by drug treatment used in this analysis was from a meta-analysis of individual participant-level data from 48 randomized trials^2^.

^a^ Extended dominance indicates larger ICER than a more effective strategy.

^b^ Dominated indicates higher cost and lower QALYs than the comparator.

# Table S6. Sensitivity analysis of the cost-effectiveness of intervention strategies based on low compliance rate

|  | All individuals | | |  | High CVD risk individuals | | |
| --- | --- | --- | --- | --- | --- | --- | --- |
|  | Costs ($) | QALYs | ICER ($/QALY) |  | Costs ($) | QALYs | ICER ($/QALY) |
| From age 40 years |  |  |  |  |  |  |  |
| No intervention | 3276.66 | 16.33 | Ref |  | 8757.99 | 15.12 | Ref |
| Salt substitution | 3445.75 | 16.36 | 6413.62 |  | 8896.00 | 15.19 | 2001.79 |
| Drug treatment | 4740.71 | 16.35 | Extended dominance^a^ |  | 9791.99 | 15.22 | Dominated^b^ |
| Salt substitution and drug treatment | 4760.91 | 16.38 | 63085.33 |  | 9605.38 | 15.28 | 7843.77 |
| From age 50 years |  |  |  |  |  |  |  |
| No intervention | 1742.56 | 13.98 | Ref |  | 2746.06 | 13.40 | Ref |
| Salt substitution | 2036.70 | 14.01 | 11069.04 |  | 3096.06 | 13.44 | 8029.53 |
| Drug treatment | 3069.03 | 14.01 | Extended dominance^a^ |  | 3993.94 | 13.46 | Extended dominance^a^ |
| Salt substitution and drug treatment | 3163.61 | 14.04 | 43061.10 |  | 4047.02 | 13.50 | 17344.27 |
| From age 60 years |  |  |  |  |  |  |  |
| No intervention | 763.12 | 10.65 | Ref |  | 926.18 | 10.45 | Ref |
| Salt substitution | 1032.28 | 10.67 | 14601.21 |  | 1222.62 | 10.47 | 12609.49 |
| Drug treatment | 1817.38 | 10.67 | Extended dominance^a^ |  | 1968.27 | 10.48 | Extended dominance^a^ |
| Salt substitution and drug treatment | 1885.53 | 10.69 | 41361.82 |  | 2030.01 | 10.50 | 26825.23 |
| From age 70 years |  |  |  |  |  |  |  |
| No intervention | 406.90 | 5.55 | Ref |  | 418.72 | 5.54 | Ref |
| Salt substitution | 568.15 | 5.56 | 22335.16 |  | 583.77 | 5.55 | 22208.13 |
| Drug treatment | 951.17 | 5.56 | Extended dominance^a^ |  | 964.81 | 5.55 | Extended dominance^a^ |
| Salt substitution and drug treatment | 979.84 | 5.56 | 54922.78 |  | 993.30 | 5.55 | 51904.13 |

SBP: systolic blood pressure; CVD: cardiovascular disease; $: Chinese Yuan; QALYs: quality-adjusted life year; ICER: incremental cost-effectiveness ratio.

^a^ Extended dominance indicates larger ICER than a more effective strategy.

^b^ Dominated indicates higher cost and lower QALYs than the comparator.

S1A. Markov decision tree for interventions


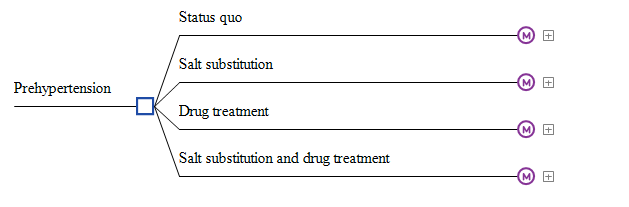


S1B. Markov decision tree for health states of each intervention


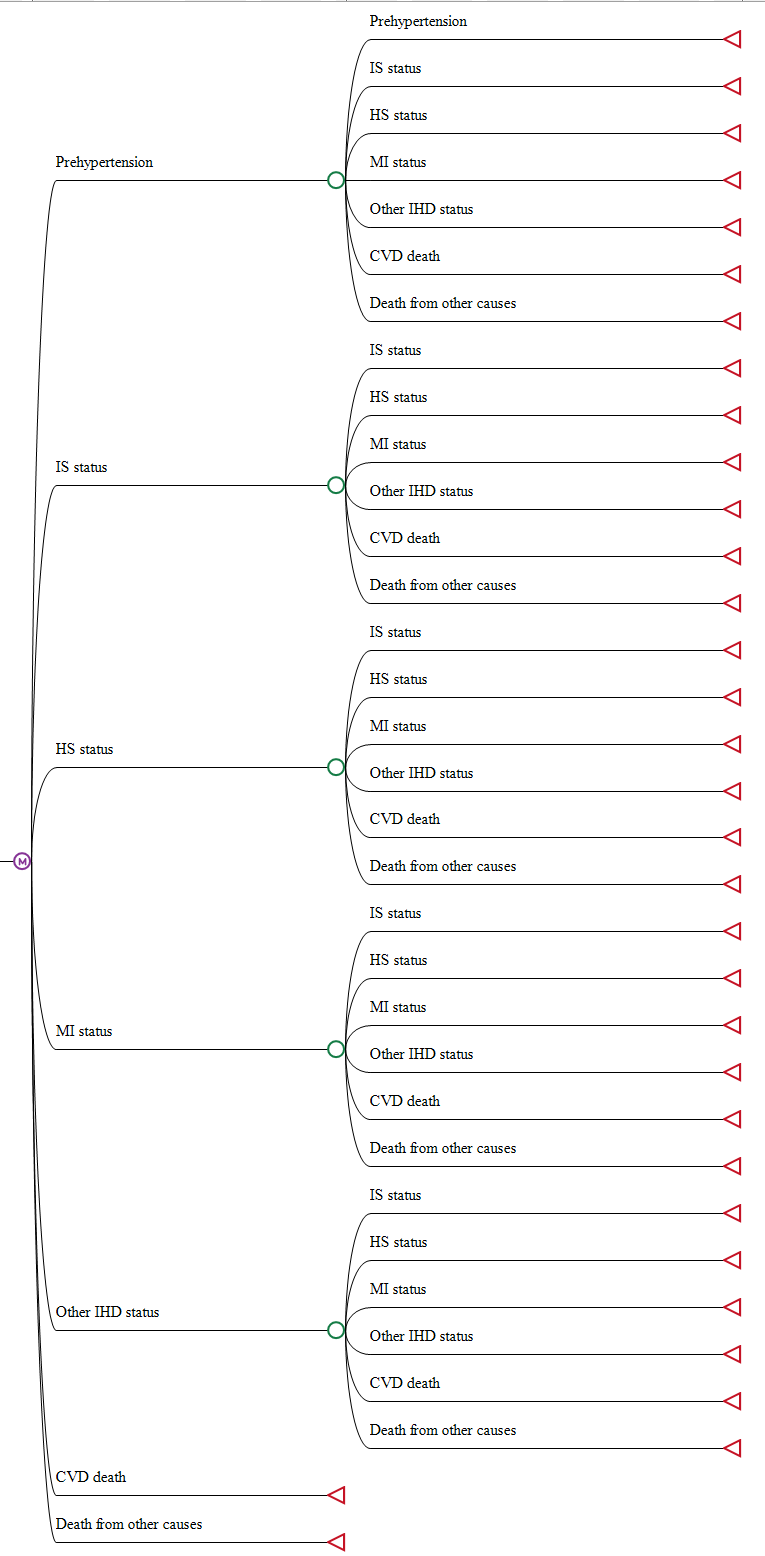


# Figure S1. Structure of the Markov model

IS: ischemic stroke; HS: hemorrhagic stroke; MI: myocardial infarction; IHD: ischemic heart disease; CVD: cardiovascular disease.
